# Rubber Hand Illusion Affects Joint Angle Perception

**DOI:** 10.1371/journal.pone.0092854

**Published:** 2014-03-26

**Authors:** Martin V. Butz, Esther F. Kutter, Corinna Lorenz

**Affiliations:** Cognitive Modeling, Department of Computer Science, Department of Psychology, Faculty of Science, Eberhard Karls University of Tübingen, Tübingen, Germany; VU University Amsterdam, Netherlands

## Abstract

The Rubber Hand Illusion (RHI) is a well-established experimental paradigm. It has been shown that the RHI can affect hand location estimates, arm and hand motion towards goals, the subjective visual appearance of the own hand, and the feeling of body ownership. Several studies also indicate that the peri-hand space is partially remapped around the rubber hand. Nonetheless, the question remains if and to what extent the RHI can affect the perception of other body parts. In this study we ask if the RHI can alter the perception of the elbow joint. Participants had to adjust an angular representation on a screen according to their proprioceptive perception of their own elbow joint angle. The results show that the RHI does indeed alter the elbow joint estimation, increasing the agreement with the position and orientation of the artificial hand. Thus, the results show that the brain does not only adjust the perception of the hand in body-relative space, but it also modifies the perception of other body parts. In conclusion, we propose that the brain continuously strives to maintain a consistent internal body image and that this image can be influenced by the available sensory information sources, which are mediated and mapped onto each other by means of a postural, kinematic body model.

## Introduction

For most of us, it is quite easy to find our way around a familiar environment. For example, we are able to find the light switch in a dark but well-known room, even without actually seeing it. In order to do so, the brain needs to be able to translate relative location estimates into suitable hand motions. To realize such translations suitable body representations and frame-of-reference transformations need to be available.

In the literature a fundamental distinction is drawn between *body image* and *body schema* representations [1], [2], [3], [4], [5]. We adhere to Gallagher's terminology, which states that a body image refers to “a system of perceptions, attitudes, and beliefs pertaining to one's own body. In contrast, a body schema is a system of sensory-motor capacities that function without awareness or the necessity of perceptual monitoring.” ([3], p. 24). In this respect, we focus on assessing aspects of the body image. In particular, we investigate if a postural, kinematic body model is utilized for maintaining an overall consistent body image across different modalities and frames of reference.

It is well known that the body image is adjusted when current sensory information about the own body is contradictory. For example, in the rubber hand illusion (RHI) [6] bodily representations are affected to certain extents due to a sensory conflict between proprioception and visuotactile information about the hand. In the original RHI experiment by Botvinick and Cohen [6], a life-sized rubber hand was placed in an anatomically plausible position while the corresponding own hand was hidden from view. During the experiment, both the real and the artificial hand were stroked with paintbrushes. When simultaneously stimulated, most of the subjects reported that they felt as if the perceived touch was generated by the monitored brush, that is, the brush that stroked the rubber hand. Furthermore, when they had to judge their hand's position with their eyes closed, their position judgments shifted toward the rubber hand. This shows that the RHI influenced aspects of the perceived body image.

Further experiments indicate that the peri-hand space appears to be mapped onto the rubber hand during RHI experiments [7]. The RHI can even lead to fearful sensations when the artificial hand is threatened [8], [9]. Armel et al. [8] suggested that the RHI might be the result of a purely bottom-up Bayesian process, in that the self-attribution of the rubber hand and a changed body image were simply constructed based on strong statistical correlations between different sensory modalities. This view was challenged by Tsakiris and Haggard [10], who showed that the drift of the unstimulated middle finger was proportional, albeit smaller, to the drift of the index and little finger when those two fingers were stroked synchronously – an effect that cannot be explained by a purely bottom-up process. The authors [10] concluded that concurrent visuotactile information was integrated into a representation of one's own body, thus allowing the generalization of the illusion to adjacent fingers. Several other recent RHI studies have investigated the effects of the illusion on reaching performance and on the feeling of body ownership [11], [12], [13], [14], [15], [16]. Most studies suggest that the RHI arises because the brain attempts to overcome the sensory conflict between visual information (that is, seeing the felt stroke on the rubber hand) and proprioceptive information (feeling the actual posture of the own arm and hand), resulting in a shift of the body image of the hand in space.

A fundamental prerequisite for successfully inducing the illusion is a plausible alignment of the fake hand in body-relative space [2], [8], [10], [17], [18], [19], [20]. When the orientations of the fake and the real hand are incongruent, for example being prone versus supine, the illusionary effects are decreased [17]. In addition, the illusionary effects are strongly weakened, or even absent, when the rubber hand is positioned far from the real hand and far from the trunk, and thus outside of the peripersonal space [20]. Generally, implausible rotations of the rubber hand can abolish the illusion [2], [10], [17], [19]. Along similar lines, subjects also reported higher ownership of the stimulated artificial hand when it was placed at an angle that was easy to mimic with the actual hand, compared to angles that are difficult to mimic [13], [21]. Finally, the agreement of the strokes simultaneously applied to the own hand and to the rubber hand is also a determinant factor for the strength of the illusion. The illusion appears to be stronger when the direction of the strokes on the rubber hand is in agreement with the strokes on the own hand in hand-relative space [22]. These results suggest that when the perceived multisensory information cannot be properly incorporated into an overall plausible body image, the strength of the illusion decreases or does not arise at all.

Seeing that the location and orientation of the rubber hand relative to the body does matter, we asked ourselves the reverse question: If the rubber hand is somewhat plausibly positioned but its relative position and orientation requires an adjustment of the internal body image beyond the hand, does the brain induce such an adjustment? In the present study we show that the modification of the body image, induced by the RHI paradigm, does indeed affect not only the locally stimulated fingers and the hand, but also estimates of the whole arm configuration. In particular, we show that the perception of the elbow joint angle is adjusted. The joint angle estimates are reported via a simple angular display, which is adjusted in accordance to the felt elbow joint configuration. The results essentially suggest that the brain attempts to continuously maintain a consistent bodily representation by incorporating all available sensory information sources, as well as knowledge about body part sizes and body kinematics. In effect, state estimates of body parts that do not stay in direct conflict with sensory information can also be indirectly affected by the RHI.

## Materials and Methods

To investigate if and to what extent bodily representations are adjusted in order to maintain an overall coherent body image, we used the RHI paradigm and extended it to ask participants to estimate the current angular configuration of their elbow joint. In addition, hand location estimates and answers to short questionnaires were gathered to determine the strength of the RHI in the participants. Additional elbow angle estimates were collected in a final baseline test to rate the general estimation accuracy for different elbow postures.

### Ethics Statement

All participants volunteered and provided written informed consent. The study was conducted in accordance with German Psychological Society (DGPs) ethical guidelines (2004, CIII), which are in accordance with the WMA declaration of Helsinki.

### Participants

Twenty-four students (six male and 18 female) participated in the experiment. Their age ranged from 18 to 40 years (mean = 22.87 years). All participants reported normal or corrected-to-normal vision and no physical limitations. One participant had an amputated index finger, so we stimulated the middle fingers. Because the participants were wearing a semitranslucent rubber glove, the appearance of the fake hand (with index finger) was only slightly strange for the participant with the amputated index finger. When excluding the data of this participant, the significant effects reported below stayed significant in all cases.

### Apparatus and Procedure

In the main experiment participants were asked to sit comfortably, relaxed, and leaning slightly back in front of a table, with the body's mid-sagittal plane facing the side of the table. The experimenter sat on the opposite side. The left elbow of the participant was placed at a particular location at the edge of the table and the left arm was oriented in a direction approximately 22° to the left of the main body axis. A box, a wooden board, and a black cape were used to cover the shoulder, the arm, and the stimulated hand of the participant, as well as the arm parts of the artificial hand. The artificial hand, which was a plaster replica of a real left lower arm and hand, was aligned with the left elbow but was placed in an orientation of 44° clockwise with respect to the real hand.

**Figure 1:**
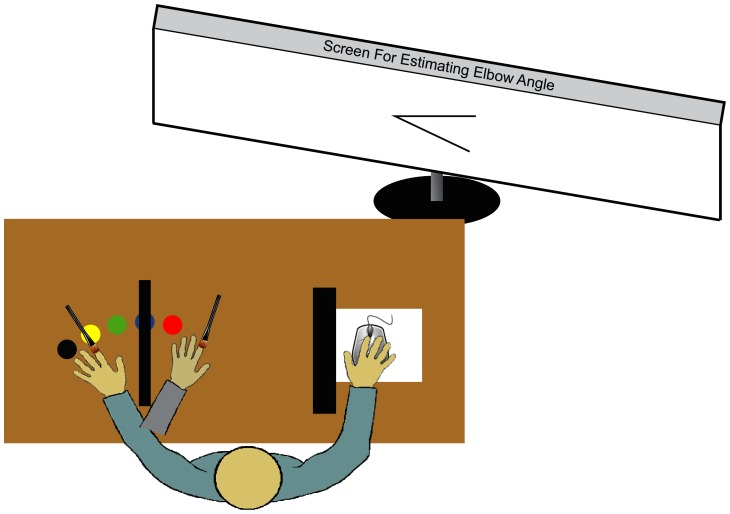
The setup of the experiment. Participants were seated comfortably, leaning slightly backwards. The own hand was hidden by a box and the entire arm was covered by a cape (not shown). The screen for the angle adjustments was easily visible without needing to move the body. The angle display was realized simply by two bars, which could be opened or closed by left and right mouse clicks. The colored dots were used for positioning the hand during the baseline test.

Both the stimulated left hand of the participants and the fake hand were covered by a semitranslucent rubber glove, which made it harder to perceive the exact details of the brush strokes. This masked, to some degree, small inconsistencies between the two strokes. The right hand of the participant was lying behind another wooden board and was used to handle the computer mouse, which was used for estimating the elbow joint angle. Figure 1 shows the experimental setup.

Three types of dependent variables were assessed: location estimates of the own hand, angular estimates of the own elbow joint angle, and a short questionnaire consisting of three questions about the strength of the RHI.

Location estimates were assessed by means of verbal reports of the location. We placed a wooden board above the fake hand, as well as the cape and the box covering the real hand. A straight and flexible tape measure was positioned on the board, each time with a different offset invisible to the participant. The tape was aligned with the board edge that faced away from the participant and the numbers on the tape (in centimeters) increased from left to right from the participants' perspective. Participants were asked to report their hand location (in centimeters) with respect to the tape measure.

Angular estimates were assessed by displaying an angle on a projection screen and asking the participants to adjust the angular display. The angle was displayed by two black bars with common origin on a white background. The distance between participants and the projected screen was approximately 1.5 meters. The bar orientations could be adjusted via left and right mouse clicks, increasing and decreasing the relative orientation angle of the two bars respectively.

Finally, three questions from Botvinick and Cohen's original paper [6] were verbally asked about the perceived illusion at the end of each trial. In particular, we used the first (Q1: ‘It seemed as if I were feeling the touch of the paintbrush in the location where I saw the rubber hand touched.’), the fourth (Q2: ‘It felt as if my (real) hand was drifting towards the right (towards the rubber hand).’) and their last question (Q3: ‘The rubber hand began to resemble my own (real) hand, in terms of shape, skin tone, freckles or some other visual feature.’) from [6]. The statements were phrased in German as follows: „Während des Experiments gab es Momente, in denen es schien, als würde…“ Q1: „… ich die Berührung des Pinsels dort spüren, wo ich die Berührung auf der Gummihand sah.“; Q2: „… sich meine echte Hand in Richtung Gummihand bewegen.“; Q3: „die Gummihand anfangen, meiner echten Hand in Form und anderen visuellen Merkmalen zu ähneln.”

The answers were reported based on a Likert scale. This psychometric scale had seven points with which the participants were asked to rate their agreement with the respective statements: (---)  =  not at all, (—)  =  very weakly, (-)  =  weakly, (0)  =  moderately, (+)  =  strongly, (++)  =  very strongly, (+++)  =  absolutely (in German: (---)  =  “überhaupt nicht”, (—)  =  “sehr schwach”, (-)  =  “schwach”, (0)  =  “mittelmäβig”, (+)  =  “stark“, (++)  =  “sehr stark”, (+++) “absolute”). While Q1 and Q3 were assessed to monitor the conscious judgment of the strength of the illusion, Q2 was assessed to identify overly compliant participants, seeing that participants typically disagree with it.

Each trial started by estimating hand position and elbow angle. To avoid angular estimation biases, the elbow angle had to be estimated twice. First, the displayed angle was initially wide and participants had to decrease its size, while the second time the angle was initially narrow, or vice versa. The stimulation followed the initial measurements. The real and false index fingers were stroked for three minutes by the experimenter with two small paint brushes. Approximately one stroke per second was applied. To manipulate the strength of the illusion, the stimulation was either congruent or incongruent. In the congruent condition the strokes were applied in the same direction; in the incongruent condition the strokes were applied in opposite directions. However, in both cases we applied similar stroke dynamics to minimize the consciously perceived perceptual difference. We expected weaker illusion effects in the incongruent condition.

While the hands were stimulated, participants were asked to keep their visual focus on the fake hand. They were further instructed not to move their upper body, arm, or hand. After the stimulation, they first estimated their elbow angle and then reckoned the hand position via the tape measure. Lastly, the answers to the short questionnaire were recorded.

Each experiment consisted of four blocks of two trials each. In each block one congruent and one incongruent stimulation trial were conducted in randomized order across blocks and participants. After each trial, participants were instructed to lift their left hand for a few seconds to decrease sequence effects across trials. After each block of two trials, participants were allowed to move and relax their arms during a break of approximately 30 seconds.

After four blocks of trials, a final baseline test was conducted. The setting was similar except that the box and boards were removed. The left elbow was placed at the same location as during the experiment, but the left hand was positioned at one of five different locations, which were marked with different colors (cf. Figure 1). The five locations were arranged in a half-circle with the midline facing away from the participant. The individual locations were separated by an angle of 11°. According to the program's instructions on the screen, participants placed their hand in the direction of one of the points, the experimenter covered the arm and hand with the cape, and the participant estimated the angle twice. The order of directions was randomized. Each direction was estimated twice.

### Summary

In sum, we recorded the angle estimation and the estimated hand position before and after hand stimulation. After each hand stimulation period and the subsequent recording of elbow angle and hand location estimates, a three-item questionnaire was conducted. In a final baseline test, we assessed a basic estimation accuracy of the elbow angle by positioning the lower arm in different orientations away from the body, recording the estimated elbow angles.

Our main hypotheses were that the elbow angle judgments should be affected by the RHI in a way similar to the hand location judgments. Due to the necessarily indirect influence of the RHI on the elbow angle perception, mediated by a postural, kinematic body model, we expected that the angular judgments should be affected less strongly. The questionnaire was given to test if the RHI was also fully consciously experienced. In addition, Q2 was asked to be able to identify willingly compliant subjects who simply confirm everything. In accordance to Botvinick and Cohen's original paper [6], we expected that the answers to Q1 and Q3 should be positively affected by the RHI, while Q2 should hardly be affected. Data analysis was performed using the SPSS 21 statistical software program.

## Results

In the final baseline test, the hand was positioned towards different locations on a circle, while the elbow position remained constant. The perceived elbow angle was estimated twice in each orientation. A repeated measures analysis of variance (ANOVA) showed that the forearm orientation was a significant factor for estimating the elbow angle (*F*(4,92)  =  48.97, *p*<.001). Participants reported wider angles for hand positions that were further from the body than for those that were closer to the body. We identified two participants whose angle estimates for the most extended and the least extended arm orientation did not differ by more than 3°. These two participants were excluded from any further analyses that concerned the angular estimations. Note, however, that the results reported below were not significantly altered by this exclusion. That is, all significant effects identified were also significant with these two participants included. The ANOVA for the twenty-two remaining participants showed similar significance (F(4,84)  =  60.08, p<.001). The mean estimates for the different arm positions were 131.3°, 123.0°, 115.2°, 109.5° and 100.4°, starting with the most extended position. These values yield an average angular change between neighboring positions of 7.7° degrees, which is less than the actual angular difference between adjacent locations (which was approximately 11°). However, these subjective estimates can be considered approximately correct because the shoulder joint orientation also contributed to the orientation of the lower arm.

To detect overly compliant subjects we further analyzed the answers to Q2. There were twelve trials in which participants very strongly or even absolutely agreed with Q2. Since no participant revealed such affirmatives more than twice, no data was excluded.

**Figure 2:**
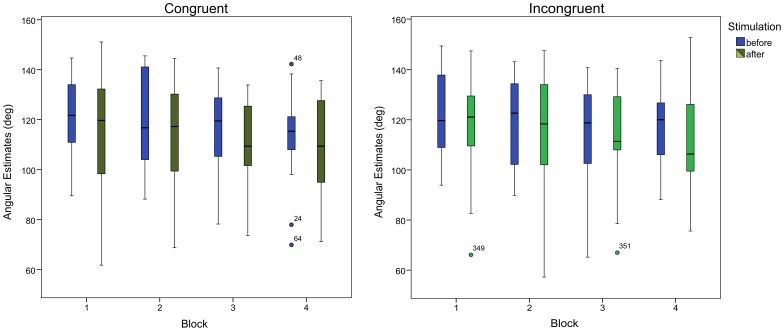
The angular estimates of the elbow joint are influenced by the RHI. After the stimulation of the hand, the angle estimate is more in agreement with the orientation of the fake hand, that is, it is smaller than before the stimulation. This effect becomes more pronounced over the blocks of the experiment. The boxplots encapsulate the medians and range from the end of the first to the end of the third quartile. The length of the whiskers approximate the 95% confidence range (1.5 times the height of the box or minimum or maximum, respectively, dependent on the data). Points denote outliers. Extreme outliers (values more than three times the height of the box) are indicated by stars.

During the main experiment, the angle estimates and hand location estimates were assessed before and after the stimulation. Repeated measurement ANOVAs were used for hand localizations and perceived elbow angles with the factors stimulation (before / after), congruency (congruent / incongruent) and block (four blocks). Due to violations of sphericity we report Greenhouse-Geisser adjusted values throughout. The ANOVA with respect to the angle estimates revealed a significant main stimulation effect (*F*(1,21) = 5.56, *p* = .028). This main effect of stimulation was due to a decrease in the angular estimates after the stimulation period, with a mean of 118.3° before stimulation versus a mean of 113.6° after stimulation. The factor block also showed a significant effect (*F*(3,63) = 6.66, *p* = .002), yielding decreasing angular estimates in the successive blocks. The main factor congruency did not reach significance (*p* = .109), nor did the interaction of the factors stimulation and congruency (*F*(1,21) = 1.96, *p* = .176). None of the other interactions yielded significant effects (all *p*≥.550). Table 1 shows the respective mean angular estimates. Figure 2 shows the results in boxplot format. Although the interaction between the factors stimulation and congruency did not reach significance, post-hoc T-tests suggest a tendency towards a stronger angle effect when the hands were stimulated congruently (*t*(21) = 2.50, *p* = .021), compared to when they were stimulated incongruently (*t*(21) = 1.71, *p* = .103).

**Table 1:** Mean elbow angle and hand location estimates in respective conditions.

Block-Averaged	congruent				incongruent			
	before		after		before		after	
angle est. (°)	118.3		112.3		118.2		114.8	
location est. (cm)	3.59		8.63		4.18		6.28	
Block-Respective	block 1				block 2			
	congruent		incongruent		congruent		incongruent	
	beforeafter	after	before	after	beforeafter	after	before	after
angle est. (°)	122.1	115.6	121.1	117.5	120.0	114.4	118.8	116.2
location est. (cm)	0.42	6.58	2.52	5.04	3.10	7.54	3.54	4.81
	block 3				block 4			
	congruent		incongruent		congruent		incongruent	
	beforeafter	after	beforeafter	after	beforeafter	after	beforeafter	after
angle est. (°)	116.9	110.1	114.9	113.8	114.3	109.1	117.9	111.8
location est. (cm)	4.85	9.54	5.69	7.23	6.00	10.85	4.98	8.02

**Figure 3:**
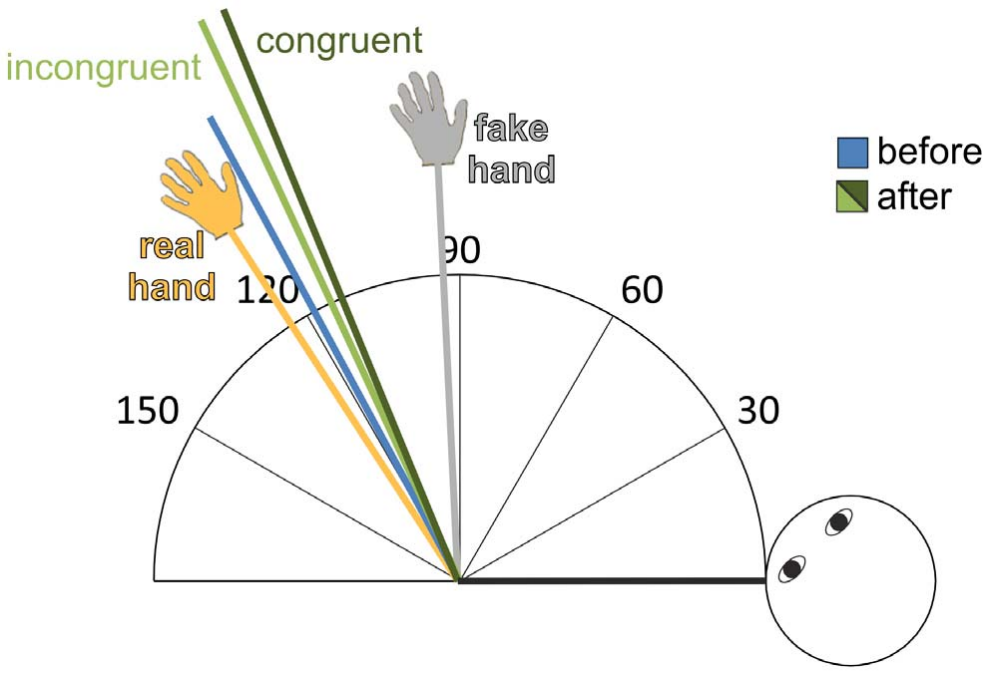
Angle estimates shift towards the fake hand. Besides the significant main effect of the shift of the angle estimates towards the rubber hand after stimulation, this shift also appears to be more pronounced in congruent stimulation trials. The joint angles corresponding to the real hand and fake hand were derived from the baseline test estimations.

Figure 3 illustrates the angle effect from a top view, plotting the average elbow angle estimates given before and after congruent and incongruent trials. To visualize the strength of the illusion, we also plot the angle estimate of the actual arm, when the rubber hand is not present, as well as an angle estimate that corresponds approximately best to the fake hand. For the angle estimate of the elbow joint angle of the actual arm we used the corresponding estimate from the baseline test. For the angle estimate that corresponds best to the fake hand, we interpolated the most inner estimate of the baseline test by another 7.7°, yielding approximately 92.7°, because the rubber hand was oriented another 11° (with respect to the table as the frame of reference, which corresponded to approximately 7.7° estimated angles as assessed in the baseline test) further towards the body (cf. Figure 1 and results of the baseline test detailed above).

**Figure 4:**
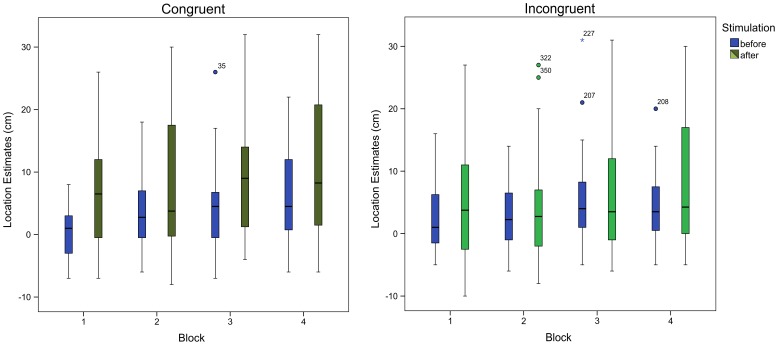
The location estimates are influenced by the RHI as expected. After the stimulation of the hands, the estimate drifts towards the artificial hand, that is, it increases. During the congruent trials the illusion is more pronounced. The block order also has an effect on the estimates.

The ANOVA analysis with respect to hand localizations revealed a significant main effect of the factor stimulation (*F*(1,23) = 9.72, *p* = .005), a significant main effect of the factor block (*F*(3,69) = 9.11, *p*<.001), and a significant main effect of the factor congruency (*F*(1,23) = 4.34, *p* = .048). Moreover, a significant interaction between the factors stimulation and congruency (*F*(1,23) = 13.23, *p* = .001) could be identified. Before stimulation the reported hand position was further away from the fake hand than after stimulation, and this difference was more pronounced for the cases when the hands were stimulated congruently. A paired T-test confirmed this assessment – the difference between hand location estimates before and after stimulation only reached significance when the hands were stimulated congruently (*t*(23) = 3.98, *p* = .001), not when they were stimulated incongruently (*t*(23) = 1.81, *p* = .084). None of the other interactions reached significance (all *p*≥.482). Tab. 1 shows the respective mean location estimates. Figure 4 shows the interactions by means of a boxplot.

**Figure 5:**
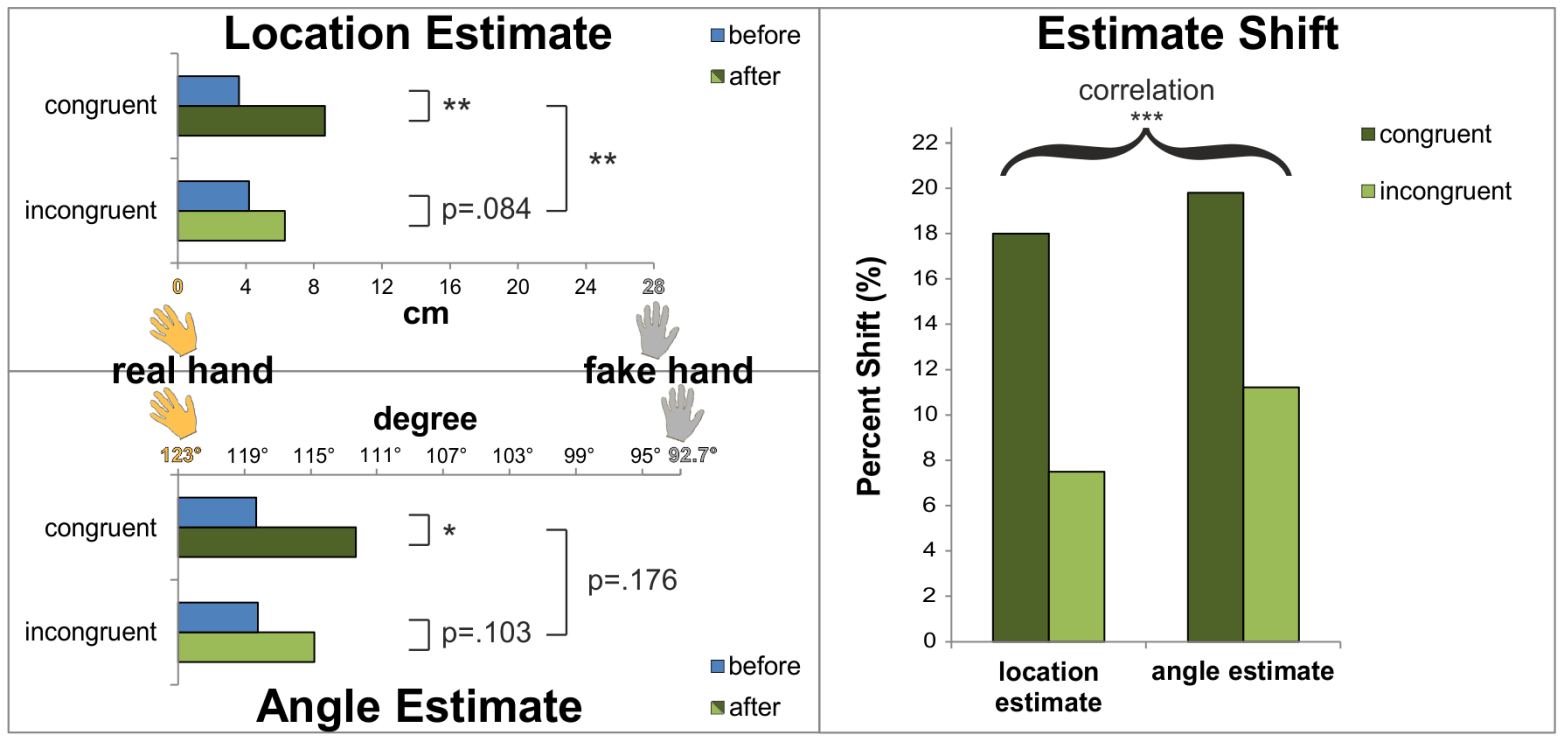
The RHI influences location estimates and angle estimates in a comparable fashion. While the statistical effects are weaker for the angle estimates, the amounts of change in location and angle estimates are comparable.

Figure 5 contrasts the induced estimation shifts towards the rubber hand recorded with respect to the elbow angle and the hand location estimates. For the location of the actual hand and of the fake hand, we used the center of the hand as the approximate location. For the elbow joint angles of the real hand and the fake hand we used the estimates of the baseline test, as explained above with respect to Figure 3. These results show a clear tendency towards similarly strong influences of the RHI on hand location estimates and on joint angle estimates.

**Figure 6:**
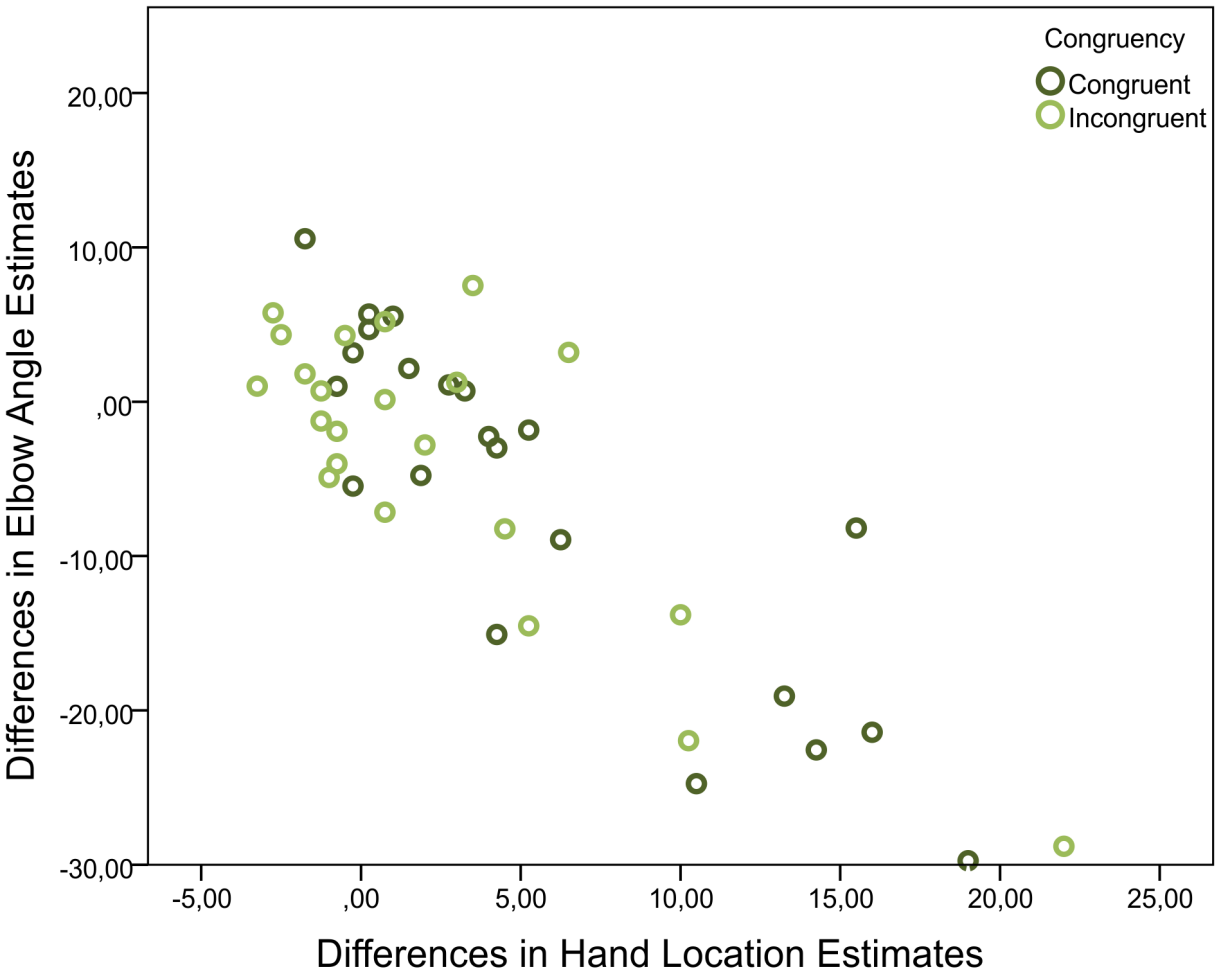
Location and angle estimates correlate significantly. Shown are the participant-respective differences in the reported hand locations and elbow angles before and after congruent and incongruent trials averaged over the four blocks.

To further statistically validate this correlation, we investigated whether the differences in the reported hand location estimates before and after stimulation correlated with the differences in the reported elbow angle estimates before and after stimulation. The correlation was high for the congruent condition (Pearson's *r*(88) = −.71, *p*<.001) as well as for the incongruent condition (Pearson's *r*(88) = −.62, *p*<.001). These results confirm that an increase in location estimates towards the rubber hand strongly correlated with a decrease in the angular estimates, which essentially corresponds to an increase in the agreement of the elbow angle with the position and orientation of the rubber hand. In order to avoid correlation effects due to the identified block dependency, the differences in the reported hand locations and elbow angles before and after congruent and incongruent trials averaged for each participant over the four blocks were also analyzed for correlation, yielding an even higher correlation (Pearson's *r*(44) = −.84, *p*<.001). Figure 6 shows this correlation in the form of a scatter plot. Overall, the correlation results suggest that when the RHI influenced the perception of the hand's location, the perception of the elbow angle configuration was equally affected.

**Figure 7:**
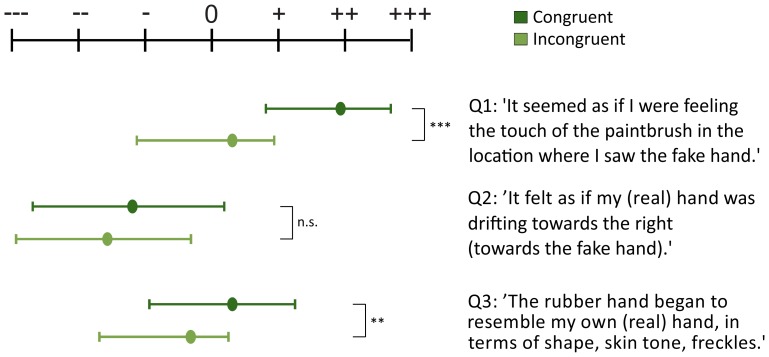
Subjective assessment of the illusion. Median and inner quartiles of the three questions after congruent and incongruent stimulation. Answer possibilities ranged on a seven-step Likert scale from agreeing ‘absolutely’(+++) to ‘not at all’(---). In the congruent condition participants agreed significantly more with Q1 and Q3 than in the incongruent condition. As expected, participants agreed the least with Q2.

We also investigated effects on the answers to the three questions asked after each trial. A repeated measurements ANOVA with the factors question, congruency, and block showed that the subjective judgments on the strength of the RHI did indeed differ with respect to the factor congruency (*F*(1,23) = 25.43, *p*<.001). The main factor block also reached significance (*F*(3,69) = 3.47, *p* = .045), as did the factor question (*F*(2,46) = 18.65, *p*<.001). Moreover, the interaction between factors question and congruency reached significance (*F*(2,46) = 7.31, *p* = .007), while no other interactions did (all *p*≥.107). In all cases, the congruent condition yielded stronger agreement with the illusion questions: Q1 in congruent condition (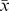 = 1.468) versus in the incongruent condition (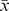 = −.021), Q2 in congruent condition (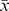 = −.990) versus in the incongruent condition (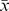 = −1.604), and Q3 in congruent condition (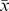 = .177) versus in the incongruent condition (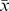 = −.672). Paired Wilcoxon Signed-Rank Tests (two-sided) to compare the effect of congruency on the answers to each question showed that only the answers to Q1 and Q3 differed significantly (Bonferroni corrected p-Values: Q1: *p*<.001; Q2: p = .12; Q3: *p* = .024), which corresponds to results found in the recent literature [23]. Figure 7 shows the answer distributions on the Likert scale in congruent and incongruent trials. With respect to block, the medians and inner quartiles of the block-respective questions showed that all questions were answered with progressively stronger agreement.

**Figure 8:**
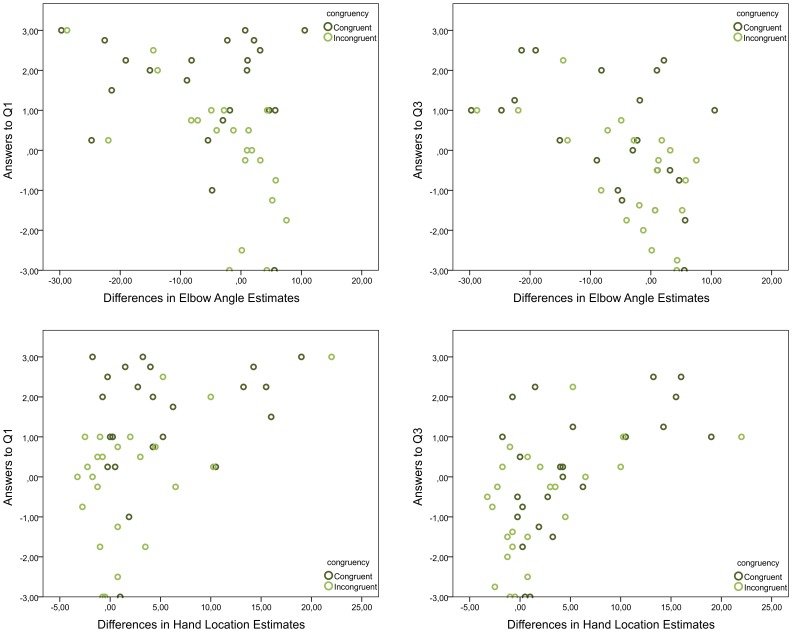
Subjective illusion assessments correlate with angle and with location estimates. The more participants agreed to Q1 or Q3, the closer their location estimates to the fake hand and the smaller their angle estimates, fostering agreement with the fake hand. Shown are the participant-respective answers (Q1: left column; Q3: right column) in congruent and in incongruent trials and the corresponding differences in angular (top row) and location (bottom row) estimates before and after stimulation averaged over the four blocks.

The similar main effects across recorded measures raised the question if the answers to the questionnaire correlate with the location and angular estimates. If anything, we expected correlations of the angular and location estimates with Q1 and Q3, while Q2 was expected not to be significantly correlated, and indeed, answers to Q1 correlated with the changes in angle estimates (Spearman's *r*(176) = −.28, *p*<.001), as did the answers to Q3 (Spearman's *r*(176) = −.35, *p*<.001). In contrast, there was no correlation with Q2 (Spearman's *r*(176) = −.06, *p* = .43). The correlations between verbal responses and differences in hand location estimates before and after stimulation yielded analogical results: Q1 (Spearman's *r*(192) = .32, *p*<.001), Q2 (Spearman's *r*(192) = .07, *p* = .33), and Q3 (Spearman's *r*(192) = .43, *p*<.001). To determine block-independent correlations, we also analyzed the answers to the questions averaged over the four blocks for congruent and incongruent trials, correlating these averages with the correspondingly averaged differences in location and angular estimates before and after stimulation. The results yielded similar correlations: answers to Q1 and Q3 correlated with the differences in angular estimates (Q1: Spearman's r(44) = −.35, p = .019, Q3: Spearman's r(44) = −.516, p<.001) but answers to Q2 did not (Q2: Spearman's r(44) = .019, p = .905). Similarly, answers to Q1 and Q3 correlated with the differences in location estimates (Q1: Spearman's r(48) = .42, p = .003; Q3: Spearman's r(48) = .54, p<.001) but answers to Q2 did not (Spearman's r(48) = .07, p = .634). Note that the slightly weaker effect strength with respect to Q1 can be explained by a ceiling effect, in which case participants fully agreed with Q1. Figure 8 visualizes the significant correlations, further discriminating between congruent and incongruent trials. In conclusion, the closer the hand's location or the elbow angle was estimated to the fake hand, the stronger the participants approved Q1 and Q3, but not necessarily Q2.

To assess if the mere presence of the fake hand had an influence on the angle estimate, we compared the angular estimates of the participants during the baseline test when the hand was positioned exactly where the hand was during the rubber hand experiment (which was the second furthest position from the own body) with the angular estimates before a stimulation trial during the experiment. Indeed, the difference reached strong significance (*t*(21) = −3.52, *p* = .002). However, this was only the case when aggregating the angular estimates before stimulation over all trials. When calculating a paired T-test with the data from only the first trial in the experiment, there was no significant difference detectable (*t*(21) = 0.09, *p* = .932). Thus, the data suggests that the presence of the artificial hand alone did not affect the perception of the elbow angle. Moreover, the data indicates that the illusion did not fully fade away until the start of the next trial, thus yielding the effect that the averaged angular estimates before all trials significantly differ from the corresponding estimates assessed in the baseline test.

The complete data set is available online at http://www.cm.inf.uni-tuebingen.de/data.

## Discussion

The RHI has attracted a lot of attention in past years. The illusion implies that even the own body is not a pre-existing, fixed entity to the brain. Instead, the brain continuously estimates the configuration, orientation, and position of its own body in the surrounding space. Providing incompatible sensory information, such as incompatible visual information in the RHI, leads to a sensory conflict and thus to a potential mislocalization of the hand and hand-relative spatial encodings. The question addressed in this study was how the brain attempts to resolve such a sensory conflict. In particular, we asked if the brain only adjusts its estimates about the body part for which a sensory conflict is present, or if the brain also adjusts the estimates of other body parts to maintain an overall consistent body image. In particular, we investigated whether only the estimate of the hand position is affected by the RHI, or if the estimate of the elbow joint angle is also affected.

The presented results suggest that the latter is the case. Angular estimates were adjusted in that the elbow angle estimate decreased, reporting a joint angle that is in better agreement with the fake hand's position and orientation. Shifts in angular estimates also strongly correlated with shifts in corresponding location estimates towards the fake hand. Thus, the changes in angular estimates appear to be affected by mechanisms that also affect the changes of the location estimates. Clearly, however, the influence on the angular estimates is less pronounced because the sensory conflict is at the hand, not at the elbow. Most likely due to this weaker, indirect influence of the illusion on the elbow angle estimates, the estimate differences in incongruent versus congruent trials did not reach significance.

The results of the short questionnaires are also in agreement. The analysis revealed stronger effects in the congruent compared to the incongruent condition for Q1 and Q3, but not for Q2 – an effect that was observed in other studies as well [6], [11], [16], [23]. More importantly, only the answers to Q1 and Q3 significantly correlated with the effects on angular and location estimates. Thus, the body image of both the elbow and the hand were adjusted in accordance with the strength of the experienced illusion, indicating once more that the illusion in our study affected hand and elbow in a similar fashion.

Taken together, the results of the presented experiment suggest that besides the direct influence on the brain's state estimation of the own hand, the illusion also affects estimates about the state of other body parts, such as the joint angle of the elbow. This shift of other body part estimates must arise indirectly via an internal, postural model of the own body. This *body model* needs to include size and kinematic knowledge of body parts, joint angle orientations, and the influence of current joint angles on the orientation and location of the joint-dependent body parts. In Gallagher's terminology [3] this body model may be part of the body image, essentially explicitly spelling out one aspect of the “beliefs pertaining to one's own body.” Similar body models have been proposed and discussed (cf. reviews in [12], [24]), in which cases they were rather associated to the body schema, employing a slightly different terminology.

When consistent, redundant sensory information from different modalities is available about a body part, the body model enables information exchange and effective information fusion, where information fusion will most likely take place in a statistically approximately optimal way [25]. If the sensory information from different modalities is not in agreement, however, it may still be fused to some degree, leading to the mislocalization of the hand. Beyond this now rather well-known mislocalization effect, our results show that the induced sensory conflict and the brain's resolution of this conflict, that is, the adjustment of the hand's localization, also leads to a corresponding adjustment of the elbow angle estimate. This latter effect must be mediated by the mentioned postural, kinematic body model, which projects the adjusted body image of the hand onto other body parts, causing their corresponding adjustment.

Seeing that in our study the RHI affected proprioceptive representations and the estimations thereof, the results stand in contrast with the model of Makin et al. [14], which suggests that only hand location estimates are affected by the RHI. The neural Modular Modality Frame model (nMMF), which was developed in our group, is able to model angular adjustments due to incorrect sensory information about the hand [26], [27]. nMMF compares incoming multi-modal sensory information over time by means of Bayesian information processing principles. A kinematic body model is used to project modal information into other modalities and frames of reference. If sensory information is in conflict, the internal body state, which is represented neurally in the involved sensory modalities, is adjusted to increase the agreement of the information in conflict [26], [27]. To maintain an overall consistent body model, nMMF then also adjusts its estimates about dependent limb orientations and joint angles [27].

In the future, we believe that the available RHI data should be modeled in a more rigorous way. Such a computational model should also take other aspects of the illusion into account, such as the dependency on the agreement of the dynamics and on the orientation of the tactile strokes. Effects on body ownership estimations as well as on tactile sensitivity should also be considered further. Other bodily illusions suggest that estimations about other body parts, such as the length of an arm limb or of the nose [28], can be also affected by conflicting sensory information. Such illusions may be modeled along similar lines. Finally, the results cannot determine at this point whether the effect on the angle estimate is a purely conscious, “imaginary” phenomenon, or if it can also affect motor control. Thus, similar to the tests respecting directional and grasping motions, which can be affected by the RHI to certain extents [1], [23], [29], [30], [31], the execution of arm movements that depend on the elbow orientation should be tested in future work.

## References

[1] Kammers MP, Kootker JA, Hogendoorn H, Dijkerman HC (2010) How many motoric body representations can we grasp? Experimental Brain Research 202: 203–212. 20039029 10.1007/s00221-009-2124-7 PMC2845887

[2] Ehrsson HH, Spence C, Passingham RE (2004) That's my hand! Activity in premotor cortex reflects feeling of ownership of a limb. Science 305: 875–877. 15232072 10.1126/science.1097011

[3] Gallagher S (2005) How the body shapes the mind. Oxford: Clarendon Press.

[4] Paillard J (1999) Body schema and body image: a double dissociation in deafferented patients. In: Gantchev GN, Mori S, Massion J, editors, Motor control, today and tomorrow, Moscow: Izdatelstvo. pp. 197−214.

[5] Haggard P, Wolpert DM (2005) Disorders of body schema. In: Freund HJ, Jeannerod M, Hallett M, Leiguarda R, editors, Higher-order motor disorders: From neuroanatomy and neurobiology to clinical neurology, Oxford: Oxford University Press. pp. 261−271.

[6] Botvinick M, Cohen J (1998) Rubber hands ‘feel’ touch that eyes see. Nature 391: 756. 9486643 10.1038/35784

[7] Farnè A, Pavani F, Meneghello F, Làdavas E (2000) Left tactile extinction following visual stimulation of a rubber hand. Brain 123: 2350–2360. 11050034 10.1093/brain/123.11.2350

[8] Armel KC, Ramachandran VS (2003) Projecting sensations to external objects: evidence from skin conductance response. Proceedings of the Royal Society of London Series B: Biological Sciences 270: 1499–1506. 12965016 10.1098/rspb.2003.2364 PMC1691405

[9] Ehrsson HH, Wiech K, Weiskopf N, Dolan RJ, Passingham RE (2007) Threatening a rubber hand that you feel is yours elicits a cortical anxiety response. Proceedings of the National Academy of Sciences 104: 9828–9833. 10.1073/pnas.0610011104 PMC1887585 17517605

[10] Tsakiris M, Haggard P (2005) The rubber hand illusion revisited: visuotactile integration and self-attribution. Journal of Experimental Psychology: Human Perception and Performance 31: 80–91. 15709864 10.1037/0096-1523.31.1.80

[11] Folegatti A, de Vignemont F, Pavani F, Rossetti Y, Farnè A (2009) Losing one's hand: visual-proprioceptive conflict affects touch perception. PLoS One 4: e6920. 19738900 10.1371/journal.pone.0006920 PMC2732904

[12] Holmes NP, Spence C (2004) The body schema and multisensory representation(s) of peripersonal space. Cognitive Processing 5: 94–105. 16467906 10.1007/s10339-004-0013-3 PMC1350799

[13] Kalckert A, Ehrsson HH (2012) Moving a rubber hand that feels like your own: dissociation of ownership and agency. *Frontiers in Human Neuroscience* 6: 40. 22435056 10.3389/fnhum.2012.00040 PMC3303087

[14] Makin TR, Holmes NP, Ehrsson HH (2008) On the other hand: Dummy hands and peripersonal space. Behavioural Brain Research 191: 1–10. 18423906 10.1016/j.bbr.2008.02.041

[15] Pavani F, Zampini M (2007) The role of hand size in the fake-hand illusion paradigm. Perception-London 36: 1547. 18265837 10.1068/p5853

[16] Rohde M, Di Luca M, Ernst MO (2011) The rubber hand illusion: Feeling of ownership and proprioceptive drift do not go hand in hand. PloS one 6: e21659. 21738756 10.1371/journal.pone.0021659 PMC3125296

[17] Austen EL, Soto-Faraco S, Enns JT, Kingstone A (2004) Mislocalizations of touch to a fake hand. Cognitive, Affective, & Behavioral Neuroscience 4: 170–181. 10.3758/cabn.4.2.170 15460923

[18] Pavani F, Spence C, Driver J (2000) Visual capture of touch: Out-of-the-body experiences with rubber gloves. Psychological Science 11: 353–359. 11228904 10.1111/1467-9280.00270

[19] Lloyd D, Morrison I, Roberts N (2006) Role for human posterior parietal cortex in visual processing of aversive objects in peripersonal space. Journal of Neurophysiology 95: 205–214. 16162829 10.1152/jn.00614.2005

[20] Preston C (2013) The role of distance from the body and distance from the real hand in ownership and disownership during the rubber hand illusion. Acta psychologica 142: 177–183. 23333877 10.1016/j.actpsy.2012.12.005

[21] Ide M (2013) The effect of “anatomical plausibility” of hand angle on the rubber hand illusion. Percept 42: 103–111. 10.1068/p7322 23678620

[22] Costantini M, Haggard P (2007) The rubber hand illusion: Sensitivity and reference frame for body ownership. Consciousness and Cognition 16: 229–240. 17317221 10.1016/j.concog.2007.01.001

[23] Zopf R, Truong S, Finkbeiner M, Friedman J, Williams MA (2011) Viewing and feeling touch modulates hand position for reaching. Neuropsychologia 49: 1287–1293. 21320514 10.1016/j.neuropsychologia.2011.02.012 PMC3086579

[24] Graziano MS, Botvinick MM (2002) How the brain represents the body: Insights from neurophysiology and psychology. In Prinz W, Hommel B, editors, Common mechanisms in perception and action: Attention and performance XIX, Oxford: Oxford University Press. pp 136−157.

[25] Ernst MO, Banks MS (2002) Humans integrate visual and haptic information in a statistically optimal fashion. Nature 415: 429–433. 11807554 10.1038/415429a

[26] Ehrenfeld S, Butz MV (2013) The modular modality frame model: Continuous body state estimation and plausibility-weighted information fusion. Biological Cybernetics 107: 61–82. 23090574 10.1007/s00422-012-0526-2

[27] Ehrenfeld S, Herbort O, Butz MV (2013) Modular neuron-based body estimation: maintaining consistency over different limbs, modalities, and frames of reference. Frontiers in Computational Neuroscience, 7. 10.3389/fncom.2013.00148 PMC3808893 24191151

[28] Lackner JR (1988) Some proprioceptive influences on the perceptual representation of the body shape and orientation. Brain 111: 281–297. 3378137 10.1093/brain/111.2.281

[29] Heed T, Gründler M, Rinkleib J, Rudzik FH, Collins T, et al (2011) Visual information and rubber hand embodiment differentially affect reach-to-grasp actions. Acta psychologica 138: 263–271. 21788001 10.1016/j.actpsy.2011.07.003

[30] Snijders HJ, Holmes NP, Spence C (2007) Direction-dependent integration of vision and proprioception in reaching under the influence of the mirror illusion. Neuropsychologia 45: 496 – 505. 10.1016/j.neuropsychologia.2006.01.003 PMC1705814 16499935

[31] Riemer M, Kleinböhl D, Hölzl R, Trojan J (2013) Action and perception in the rubber hand illusion. Experimental Brain Research 229: 383–393. 23307154 10.1007/s00221-012-3374-3

