# Correction: Rubber Hand Illusion Affects Joint Angle Perception

**DOI:** 10.1371/journal.pone.0102852

**Published:** 2014-07-09

**Authors:** The PLOS ONE Staff

There are errors in Table 1. The publisher apologizes for these errors. The authors have provided a corrected version of Table 1, which can be viewed here.

**Table1:** Mean elbow angle and hand location estimates in respective conditions.

Block-Averaged	congruent				incongruent			
	before		after		before		after	
angle est. (°)	118.3		112.3		118.2		114.8	
location est. (cm)	3.59		8.63		4.18		6.28	
**Block-Respective**	**block 1**				**block 2**			
	congruent		incongruent		congruent		incongruent	
	before	after	before	after	before	after	before	after
**angle est. (°)**	122.1	115.6	121.1	117.5	120.0	114.4	118.8	116.2
**location est. (cm)**	0.42	6.58	2.52	5.04	3.10	7.54	3.54	4.81
	**block 3**				**block 4**			
	congruent		incongruent		congruent		incongruent	
	before	after	before	after	before	after	before	after
**angle est. (°)**	116.9	110.1	114.9	113.8	114.3	109.1	117.9	111.8
**location est. (cm)**	4.85	9.54	5.69	7.23	6.00	10.85	4.98	8.02
